# The effects of AI second opinions on collaborative confidence and decision-making: evidence from a controlled study of postgraduate vocal accompanists

**DOI:** 10.3389/fpsyg.2026.1862379

**Published:** 2026-07-21

**Authors:** Tong Zhu, Li Xia

**Affiliations:** Keimyung University, Daegu, Republic of Korea

**Keywords:** decision-making style, human–AI collaboration, music education, performance anxiety, self-efficacy

## Abstract

**Background:**

Artificial intelligence (AI) is increasingly integrated into higher education, yet empirical evidence remains limited on how AI-generated “second opinions” influence psychological and cognitive outcomes in artistic training contexts.

**Objective:**

This study investigates the effects of AI “second opinions” on collaborative self-efficacy, music performance anxiety, and decision-making style among postgraduate students specialising in vocal accompaniment.

**Results:**

The experimental group showed significantly higher posttest self-efficacy than the control group (*p* = 0.026) and a significant within-group increase (*p* = 0.007). Music performance anxiety was significantly lower in the experimental group compared with the control group (*p* = 0.013), with a significant within-group reduction (*p* = 0.007). No significant differences were found in decision-making style between or within groups (all *p* > 0.05).

**Conclusion:**

AI “second opinions” can effectively enhance self-efficacy and reduce performance anxiety in postgraduate music accompaniment training. However, they do not significantly alter decision-making styles in the short term, suggesting that AI primarily functions as an augmentative tool supporting human interpretative agency rather than reshaping cognitive patterns.

## Introduction

In the third decade of the 21st century, artificial intelligence (AI) has evolved from a peripheral technological concept into a transformative force that is reshaping human cognitive patterns, social practices, and cultural production. Its penetration into the arts has been particularly rapid and far-reaching, not only continually challenging the traditional philosophical foundations of creativity, authorship, and aesthetic value (Boden, 2016; Mercier-Laurent, 2020), but also increasingly intervening at an unprecedented depth in the micro-level decision-making processes of artistic creation, performance, and education. Music, as an art form that is highly dependent on intuition, emotional resonance, and immediate interpersonal interaction, is undergoing a structural transformation driven by AI technologies. From AI composition, such as Google’s Magenta project, and intelligent arrangement to real-time accompaniment generation, these technological tools are increasingly moving beyond their auxiliary roles and becoming indispensable co-creators for musicians (Dao, 2023; Salma et al., 2025; Szostak, 2025; Wang, 2025; Xu et al., 2026). However, amid rapid technological advancement, a core question remains underexplored in empirical research. When AI evolves from a passive tool into an intelligent entity capable of offering professional opinions, how does it influence the development of confidence and decision-making strategies among human artists, particularly postgraduate students who are at a critical stage of professional development and whose professional identities are still being formed, in advanced collaborative settings? This study focuses on vocal coaching, a highly specialised field that relies on nuanced interpersonal judgement and deep trust, and aims to systematically examine the effects of AI as a Second Opinion on collaborative confidence and decision-making strategies among postgraduate vocal accompanists.

Vocal coaching is far more than piano accompaniment; it is a high-level collaborative art form that integrates musical text analysis, multilingual vocal guidance, historical stylistic interpretation, psychological support, and real-time artistic co-creation (Yan, 2025). In this process, the vocal coach must establish a relationship of trust with the singer, grounded in mutual respect and deep understanding, in order to jointly explore the emotional core, technical details, and aesthetic expression of the work. Every minute decision, whether concerning tempo, dynamics, phrasing, or timbre, arises from a complex negotiation between both parties, informed by their respective experience, intuition, cultural understanding, and immediate feedback. This decision-making process is characterised by uncertainty, subjectivity, and contextual dependence, which also constitute its distinctive artistic appeal. However, for postgraduate students who are transitioning towards becoming independent artists, this inherent ambiguity presents a significant learning challenge. Due to limited authoritative practical experience, they often experience deep self-doubt regarding their professional judgements, which in turn constrains their initiative, voice, and confidence within collaborative interactions (Kenny et al., 2004; Musgrave, 2023; McCormick and McPherson, 2003). Traditional teaching models largely rely on the tutor’s first opinion, which represents a unidirectional and authoritative mode of knowledge transmission. Although this approach provides clear guidance, it may also risk constraining students’ critical thinking, independent decision-making abilities, and the development of their individual artistic voice (Shepherd et al., 2021; Krakowski et al., 2026; Iku-Silan et al., 2023).

In recent years, the concept of an AI second opinion has gained increasing attention in high-risk and complex decision-making domains such as medical diagnosis and financial investment (Abdulnour et al., 2025; Topol, 2019; Xu et al., 2024). Its core premise is to introduce an independent intelligent system based on large-scale data and algorithmic models to complement, rather than replace, human expert judgement. This approach aims to provide diverse perspectives, reduce cognitive biases such as confirmation bias, broaden decision-makers’ perspectives, and ultimately enhance both the confidence and robustness of their decisions (Duan et al., 2019; Trunk et al., 2020; Magni and Baroncelli, 2025). Extending this interdisciplinary paradigm to the arts, particularly to the highly subjective and complex collaborative context of vocal accompaniment, carries significant theoretical and practical implications. Contemporary AI systems, especially those based on deep learning for Music Information Retrieval (MIR) and generative modelling, are capable of conducting multidimensional and objective analyses of musical scores and audio signals with a level of speed and precision that exceeds human perceptual capacity. Moreover, by learning from extensive databases of historical performances, these systems can provide stylistic and technical performance suggestions (Liu and Zhao, 2024; Le et al., 2025; Typke, 2021). Such suggestions are not intended to offer a single authoritative answer, but rather function as a verifiable and discussable second opinion that provides postgraduate students with an external frame of reference for reflecting on their initial decisions, a role that aligns with emerging evidence on the transformative potential of generative AI in interdisciplinary collaborative learning environments (Xia et al., 2026).

Although existing research has begun to examine the application of AI in music education, it has primarily focused on basic skill training, such as pitch and rhythm correction (Li, 2026; Tejada and Fernández-Villar, 2023), or compositional assistance, such as melody and harmony generation (Herremans et al., 2015). Research addressing how AI influences deeper psychological and cognitive processes in advanced musical collaboration, particularly in relation to postgraduate-level decision-making confidence, metacognitive development, and interaction dynamics with human collaborators, remains scarce, despite growing concern regarding whether AI tools empower learners or foster cognitive dependency, particularly in domains requiring high levels of interpretative judgement (Peng et al., 2026). Purwanto (2025) emphasises that the effective integration of AI in creative contexts lies not in replacing human creative agency, but in empowering it. Similarly, Duan et al. (2019) highlight that successful human-AI collaboration depends on clearly defining the role of AI and leveraging complementary cognitive strengths between humans and intelligent systems. These perspectives provide an important theoretical foundation for the present study, yet they require systematic empirical validation within complex music education contexts.

Grounded in the Human-AI Collaborative Augmentation (HACA) framework—which posits three core dimensions: (1) AI as structured external reference (providing data-driven, multi-perspective information to scaffold human judgement); (2) human interpretative agency (the learner retains ultimate decision-making authority and interpretative responsibility); and (3) augmentation rather than replacement (the human-AI interaction expands cognitive resources rather than substituting human capability)—the central hypothesis of this study is that the introduction of an AI second opinion can enhance the decision-making confidence of postgraduate vocal accompaniment students in collaborative settings by providing objective, diverse, and data-driven reference information. In this study, decision-making confidence is operationalised as self-efficacy and reduced performance anxiety. At the same time, the intervention is expected to encourage the adoption of more reflective and integrative decision-making strategies. Specifically, by reducing decision-related anxiety associated with limited experience, AI support may encourage students to express their perspectives more proactively in collaborative interactions. In addition, when engaging with structured AI-generated suggestions, students may move beyond reliance on tutor authority or intuitive judgement alone, and instead develop the ability to integrate emotional intuition and cultural understanding with data-driven analysis and pattern recognition. This process may contribute to the emergence of an augmented decision-making model grounded in human-AI collaborative augmentation.

The significance of this study is twofold. At the theoretical level, it addresses a critical gap in the literature by examining higher-order collaborative decision-making and psychological mechanisms in AI-supported music education, while integrating perspectives from Human-Computer Interaction and decision science into music pedagogy. At the practical level, the study aims to contribute to the development of a responsible and empowerment-oriented paradigm of human-AI collaborative augmentation in arts education. As leading music institutions increasingly adopt AI technologies (Zhejiang Conservatory of Music, 2025), it becomes essential to clarify how such technologies can support, rather than diminish, the core competencies of human artists. These competencies include aesthetic judgement grounded in empathy, cultural knowledge, and historical awareness. The findings of this study are expected to provide empirical guidance for educators, curriculum designers, and AI developers in the effective and ethical integration of AI into postgraduate training. Ultimately, the goal is to support the development of a new generation of musicians who are capable of engaging with advanced technologies while maintaining artistic sensitivity, humanistic awareness, and independent critical thinking.

## Research methods

### Research subjects

This study used a combination of convenience and purposive sampling to recruit full-time Master’s students specialising in vocal accompaniment from the music faculties of three “Double First-Class” universities in China (University A, University B, and University C). The inclusion criteria were as follows: (1) having completed the core courses in the vocal accompaniment programme and having entered the preparation stage for their degree recital; (2) possessing at least 2 years of experience in piano performance; (3) having no history of severe performance anxiety; and (4) voluntarily participating and signing an informed consent form. To ensure the homogeneity and representativeness of the sample, students who had majored in non-keyboard instruments or non-music disciplines at the undergraduate level were excluded.

An *a priori* power analysis conducted using G*Power 3.1 software, with an effect size (f) of 0.25, a type I error probability (*α*) of 0.05, and a statistical power (1 − *β*) of 0.80, indicated a minimum sample size of *n* = 128. Taking into account potential dropouts during the experiment, the final target sample size was set at 150 participants, who were randomly assigned to an experimental group (*n* = 75) and a control group (*n* = 75). A total of 178 students were invited to participate, of whom 150 agreed and completed all procedures (response rate = 84.3%). To assess potential selection bias, we compared the demographic profiles of participants and non-participants; no significant differences were found in gender, age, or years of piano experience, suggesting that the sample was broadly representative of the eligible population. Basic demographic information, including gender, age, undergraduate institution, major, and years of piano playing experience, was collected via a questionnaire prior to the experiment in order to control for potential confounding variables in subsequent analyses of covariance (ANCOVA).

### Research instruments

#### General self-efficacy scale (GSES)

To measure participants’ general self-efficacy, this study used the General Self-Efficacy Scale (GSES) developed by Schwarzer and Jerusalem (1995). The scale consists of 10 items rated on a four-point Likert scale, with higher scores indicating stronger self-efficacy. The Chinese version revised by Wang et al. (2001) was adopted. Previous studies have demonstrated high internal consistency and strong construct validity, supporting its suitability for Chinese adult populations. Although the GSES primarily captures individual self-efficacy beliefs, it serves as a proxy for collaborative confidence in this study insofar as enhanced general self-efficacy has been consistently linked to greater initiative and engagement in interpersonal and collaborative contexts (Bandura, 1997). In this study, the GSES was used to assess participants’ baseline and post-intervention levels of collaborative confidence.

#### Music performance anxiety inventory (MPAI)

Music performance anxiety (MPA) was measured using the Music Performance Anxiety Inventory (MPAI). This multidimensional scale assesses cognitive, physiological, and behavioural aspects of anxiety in performance contexts. The Chinese version validated by Pan et al. (2025) was adopted, demonstrating strong reliability with Cronbach’s *α* values exceeding 0.85. The instrument uses a seven-point Likert scale, with higher scores indicating higher levels of anxiety. In this study, the MPAI was used as an inverse indicator of collaborative confidence.

#### Decision style inventory (DSI)

Decision-making style (DMS) was assessed using the Decision Style Inventory (DSI) developed by Allinson and Hayes (1996). Based on dual-process theory, the instrument measures intuitive and analytical dimensions of decision-making. The scale consists of 38 items rated on a five-point scale. As no standardised Chinese version was available, a forward-backward translation procedure was conducted. Two bilingual experts independently translated the instrument, followed by reconciliation and cultural adaptation by a third expert. A pilot test was conducted prior to formal data collection to assess clarity and internal consistency. In the pilot sample (*n* = 30), the Chinese version of the DSI demonstrated acceptable internal consistency: Cronbach’s *α* = 0.78 for the intuitive subscale and *α* = 0.81 for the analytical subscale. In the main study sample (*n* = 150), the corresponding values were α = 0.80 and α = 0.83 respectively, confirming satisfactory reliability of the adapted instrument.

#### State collaborative confidence self-assessment (SCCS)

To directly assess participants’ situational collaborative confidence at post-test, this study administered the State Collaborative Confidence Self-Assessment (SCCS), a six-item scale developed specifically for this study. Items were rated on a five-point Likert scale (1 = strongly disagree, 5 = strongly agree), with higher scores indicating greater confidence in collaborative decision-making. Sample items include “I feel confident in expressing my musical judgements during collaboration” and “I trust my ability to make effective decisions in a vocal accompaniment partnership.” In the main study sample, the SCCS demonstrated good internal consistency (Cronbach’s α = 0.86), supporting its use as a measure of collaborative confidence in the present context.

#### AI intervention system

The AI intervention was implemented using the NetEase Tianyin platform, specifically its intelligent arrangement module. Participants in the experimental group first created a MIDI-based accompaniment for a designated art song using digital audio workstation software. The melody track was then extracted and processed by the AI system, which generated alternative accompaniment suggestions based on learned stylistic patterns. These AI-generated outputs were used as a second opinion, providing additional reference points in terms of harmonic structure, rhythmic texture, and expressive interpretation. Rather than offering a single correct solution, the system presented multiple stylistic possibilities derived from large-scale musical data. Participants were instructed to compare their initial compositions with the AI-generated suggestions and revise their work accordingly. This process was designed to encourage reflective decision-making by integrating human artistic intuition with data-driven analysis, thereby supporting the development of collaborative confidence and decision-making style.

#### Research procedure

This study adhered to standard ethical procedures for experimental research, and all protocols were approved by the Henan University Ethics Committee (Approval No.: 2025–0817). A pre-test and post-test design with two groups (experimental and control) was adopted. All participants first completed an online pre-test questionnaire 1 week before the experiment. The questionnaire included the General Self-Efficacy Scale (GSES), the Decision-Making Style Scale, and the Music Performance Anxiety Inventory (MPAI), in order to establish baseline measures. Participants were then randomly assigned to either the experimental group or the control group using a computer-generated random number sequence. Allocation was concealed from participants until the day of the experiment; an independent research assistant not involved in data collection performed the assignment. Participants were directed to separate laboratory settings.

**Figure 1:**
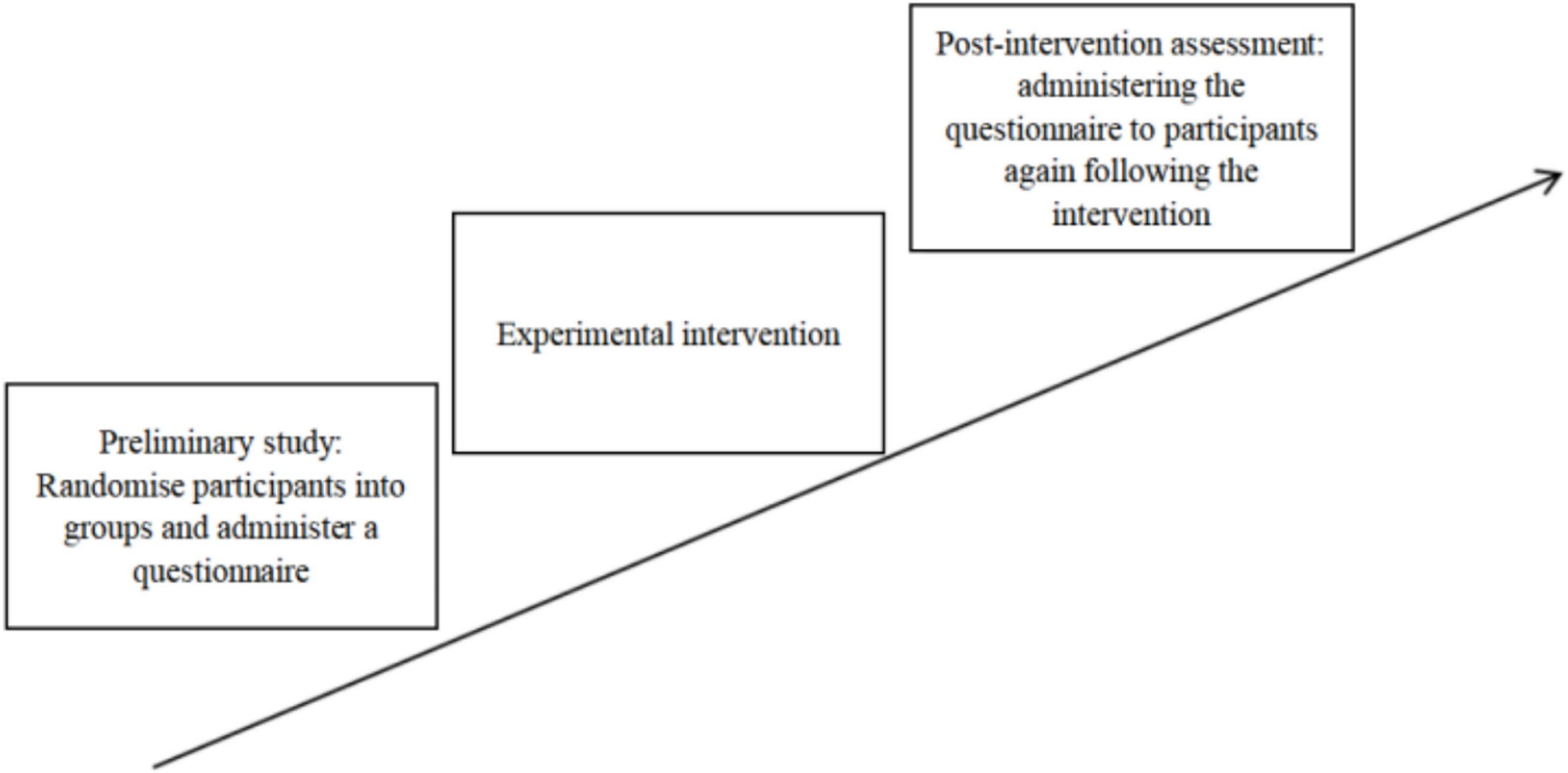
Research workflow.

Both groups completed the same standardised vocal accompaniment simulation task, which involved creating an individual piano accompaniment MIDI file in a digital audio workstation based on a designated art song score. The procedure differed after the initial draft. Participants in the experimental group uploaded the melody track to the “NetEase Tianyin” AI platform and obtained accompaniment suggestions generated by its intelligent arrangement module. They were then asked to review these suggestions and revise their original draft accordingly. Participants in the control group revised their work independently, relying only on their own knowledge and experience (no additional external feedback, reflection prompts, or reference materials were provided to the control group, so that any differences between groups can be attributed to the presence versus absence of the AI second opinion rather than to general information input) (the experimental procedure is shown in Figure 1).

Immediately after completing the task, all participants completed the post-test questionnaires, including the Decision-Making Style Inventory (DSI), the General Self-Efficacy Scale (GSES), the Music Performance Anxiety Inventory (MPAI), and the State Collaborative Confidence Self-Assessment (SCCS). In addition, three manipulation check items were administered to experimental group participants to verify that they had engaged with the AI output as a ‘second opinion’ rather than as a definitive answer: (1) “I viewed the AI suggestions as one perspective among several possibilities” (*M* = 3.81, SD = 0.74); (2) “I felt that the AI output complemented rather than replaced my own judgement” (*M* = 3.45, SD = 0.82); and (3) “I used the AI suggestions as a reference point for reflection rather than as the correct answer” (*M* = 3.67, SD = 0.79). All three items were rated above the scale midpoint, confirming that participants perceived the AI system as providing a second opinion. The entire procedure lasted approximately 90 min. The primary statistical analysis was a 2 (Group: experimental vs. control) × 2 (Time: pre-test vs. post-test) mixed-design analysis of variance (ANOVA), which provides a direct test of the Group × Time interaction as the key index of intervention efficacy. Supplementary between-group and within-group *t*-tests were conducted to characterise the direction and magnitude of effects. To control for the familywise error rate across multiple comparisons, a Bonferroni correction was applied (*α* adjusted to 0.017 for three primary outcomes). Effect sizes are reported as Cohen’s *d* (for *t*-tests) and partial η^2^ (for ANOVAs), along with 95% confidence intervals. An additional ANCOVA was performed to confirm the robustness of the intervention effects after controlling for pre-test scores. The data were analysed using SPSS 28.0.

## Results

### Homogeneity tests

Before analysing the intervention effects, this study examined whether the experimental and control groups differed in demographic characteristics and baseline psychological variables. As shown in Table 1, independent samples *t*-tests showed no significant differences between the two groups in age (*t*(148) = 0.496, *p* = 0.621), years of piano study (*t*(148) = 0.380, *p* = 0.704), pre-test self-efficacy (*t*(148) = 0.148, *p* = 0.882), or pre-test decision-making style (*t*(148) = 0.283, *p* = 0.777). The between-group difference in pre-test performance anxiety approached statistical significance (*t*(148) = 1.889, *p* = 0.061). However, the effect size was small and the 95% confidence interval included zero, suggesting that the difference was not meaningful in practical terms. Results from the Pearson chi-square test showed no significant difference in gender distribution between the two groups (*χ*^2^ = 3.236, *p* = 0.072). Taken together, these results indicate that the two groups were comparable in terms of key background characteristics and baseline psychological states, which supports subsequent causal analysis.

**Table 1:** Homogeneity tests between experimental and control groups.

Variable	Group	*n*	*M*	*SD*	*t*/*χ*^2^	*df*	*p*
Age	Experimental	75	25.16	1.97	0.496	148	0.621
	Control	75	25	1.98			
Years of piano study	Experimental	75	14.85	2.06	0.38	148	0.704
	Control	75	14.73	1.79			
Pre-test self-efficacy	Experimental	75	2.476	0.354	0.148	148	0.882
	Control	75	2.468	0.305			
Pre-test performance anxiety	Experimental	75	4.07	0.312	1.889	148	0.061
	Control	75	3.966	0.357			
Pre-test decision-making style	Experimental	75	2.991	0.234	0.283	148	0.777
	Control	75	2.98	0.205			
Gender	Male	71	—	—	3.236	1	0.072
	Female	79	—	—			

Continuous variables were analysed using independent samples t-tests; gender distribution was analysed using Pearson chi-square test.

#### Analysis of intervention effects

To examine the effects of the AI-assisted collaborative training intervention, a 2 (Group) × 2 (Time) mixed-design ANOVA was conducted as the primary analysis, followed by between-group comparisons of post-test scores and within-group changes from pre-test to post-test as supplementary analyses. The mixed ANOVA revealed a significant Group × Time interaction for self-efficacy (*F*(1, 148) = 7.84, *p* = 0.006, η^2^ = 0.050) and for performance anxiety (*F*(1, 148) = 6.92, *p* = 0.009, η^2^ = 0.045), indicating that changes over time differed between the experimental and control groups for both outcomes. No significant Group × Time interaction was found for decision-making style (*F*(1, 148) = 0.38, *p* = 0.538, η^2^ = 0.003).

**Figure 2:**
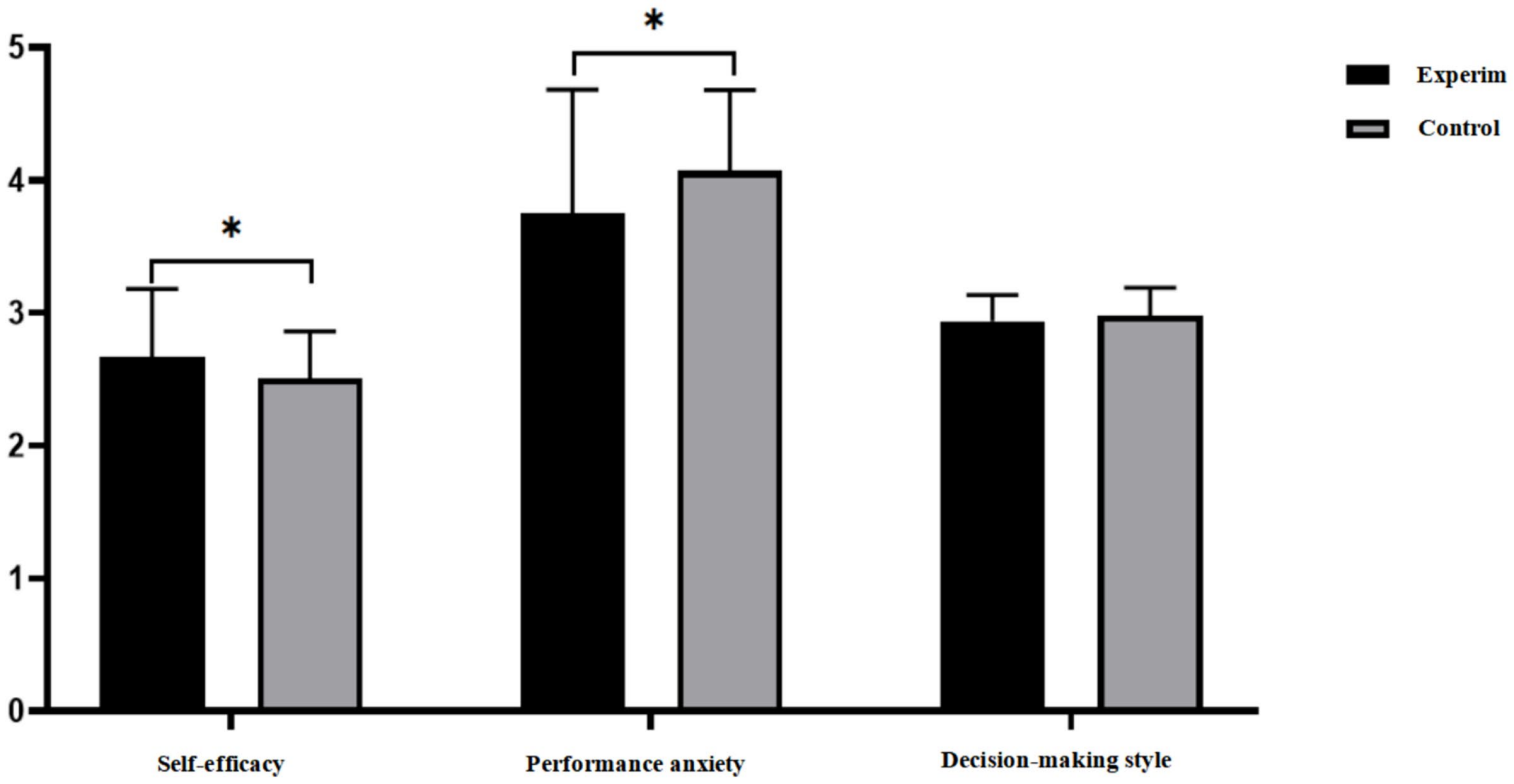
Independent samples *t*-test of post-test scores between the experimental group and the control group.

The between-group results indicated that (Table 2 and Figure 2), after the intervention, the experimental group scored higher than the control group on self-efficacy (M₁ = 2.67, SD₁ = 0.51; M₂ = 2.51, SD₂ = 0.35; *t*(148) = 2.243, *p* = 0.026, *d* = 0.37, 95% CI [0.05, 0.70]), suggesting an improvement in participants’ self-efficacy. The experimental group also reported lower levels of performance anxiety compared with the control group (M₁ = 3.75, SD₁ = 0.93; M₂ = 4.07, SD₂ = 0.61; *t*(148) = −2.509, *p* = 0.013, *d* = −0.41, 95% CI [−0.74, −0.08]), indicating a reduction in music performance anxiety. No significant difference was found between the two groups in decision-making style at post-test (*t*(148) = −1.358, *p* = 0.176, *d* = −0.22, 95% CI [−0.54, 0.10]).

**Table 2:** Between-group comparisons of post-test scores.

Variable	Group	*n*	*M*	*SD*	*t*	*df*	*p*	95% CI
Self-efficacy	Experimental	75	2.67	0.51	2.243	148	0.026	[0.019, 0.303]
	Control	75	2.51	0.35				
Performance anxiety	Experimental	75	3.75	0.93	−2.509	148	0.013	[−0.577, −0.069]
	Control	75	4.07	0.61				
Decision-making style	Experimental	75	2.94	0.20	−1.358	148	0.176	[−0.110, 0.020]
	Control	75	2.98	0.21				

Regarding the post-test confidence measures, the self-efficacy results reported above are based on the GSES. Results from the State Collaborative Confidence Self-Assessment (SCCS), administered only at post-test, showed that the experimental group (*M* = 3.92, SD = 0.61) reported significantly higher collaborative confidence than the control group (*M* = 3.54, SD = 0.58; *t*(148) = 3.74, *p* < 0.001, *d* = 0.64, 95% CI [0.30, 0.98]), providing convergent evidence that the AI second opinion enhanced situational collaborative confidence.

**Figure 3:**
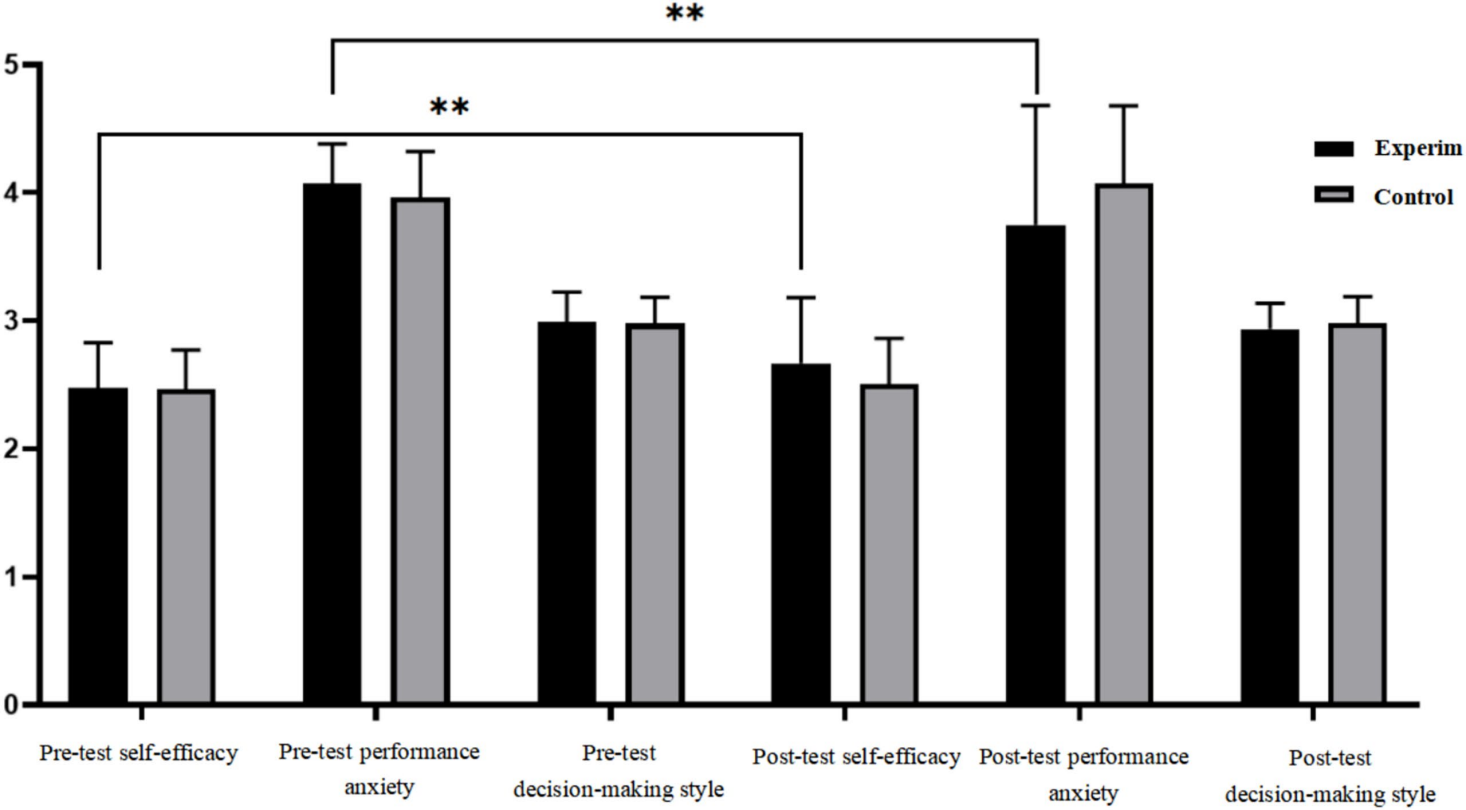
Paired-sample *t*-test of pre-test to post-test changes between the experimental group and the control group.

The within-group analyses showed changes over time (Table 3 and Figure 3). In the experimental group, self-efficacy increased from pre-test to post-test (Δ*M* = 0.193, *t*(74) = −2.786, *p* = 0.007, *d* = −0.32, 95% CI [−0.56, −0.08]), while performance anxiety decreased (Δ*M* = −0.321, *t*(74) = 2.793, *p* = 0.007, *d* = 0.32, 95% CI [0.09, 0.56]). By comparison, the control group showed no statistically significant changes in any of the variables (all *p* > 0.05). Supplementary ANCOVAs controlling for pre-test scores confirmed these patterns: the effect of group on post-test self-efficacy remained significant (*F*(1, 147) = 5.21, *p* = 0.024, η^2^ = 0.034), as did the effect on post-test performance anxiety (*F*(1, 147) = 6.08, *p* = 0.015, η^2^ = 0.040), supporting the robustness of the main findings. For the decision-making style outcome, the ANCOVA also yielded a non-significant group effect (*F*(1, 147) = 1.87, *p* = 0.173). Overall, the intervention was associated with higher self-efficacy and lower performance anxiety, but not with changes in decision-making style.

**Table 3:** Within-group comparisons of pre-test and post-test scores.

Variable	Group	Pre-test *M* (SD)	Post-test *M* (SD)	Mean difference (Post−Pre)	*t*	*df*	*p*
Self-efficacy	Experimental	2.476 (0.354)	2.669 (0.513)	0.193	−2.786	74	0.007
	Control	2.468 (0.305)	2.508 (0.354)	0.040	−0.687	74	0.494
Performance anxiety	Experimental	4.070 (0.312)	3.749 (0.933)	−0.321	2.793	74	0.007
	Control	3.966 (0.357)	4.072 (0.608)	0.105	−1.202	74	0.233
Decision-making style	Experimental	2.991 (0.234)	2.938 (0.198)	−0.053	1.404	74	0.165
	Control	2.980 (0.205)	2.983 (0.207)	0.002	−0.073	74	0.942

Given the non-significant DSI results (*p*-values of 0.165 and 0.942 for within-group changes in the experimental and control groups, respectively), a post-hoc power analysis was conducted. Assuming the originally specified effect size (*f* = 0.25) and the obtained sample size (*n* = 75 per group), the achieved power for the DSI within-group comparison was estimated at 0.51, indicating that the study was underpowered to reliably detect moderate effects in this outcome. Furthermore, a Bayesian factor analysis (BF₁₀ = 0.31) provided moderate evidence in favour of the null hypothesis, suggesting that the absence of a significant DSI effect is more likely to reflect a genuinely small effect rather than insufficient power alone. Taken together, these analyses caution against over-interpreting the null DSI result as strong evidence that decision-making style is unaffected by the intervention.

## Discussion

This study investigates the impact of “AI second opinions” on the collaborative confidence and decision-making strategies of postgraduate vocal accompaniment students in advanced collaborative settings. Based on a controlled experimental design, the findings show that the AI intervention system built on the “NetEase Tianyin” platform was associated with improved participants’ self-efficacy and reduced their levels of performance anxiety. At the same time, no significant changes were observed in individual decision-making styles along the intuition-analysis dimension over the short term. These results provide preliminary empirical support for the application of AI in arts education, while also pointing to the underlying mechanisms, limitations, and possible directions for further refinement of the Human-AI Collaborative Augmentation paradigm in complex and highly subjective contexts.

### AI as a catalyst for confidence: empowerment rather than replacement

The most salient finding concerns the role of AI second opinions as a source of support for professional confidence. In the experimental group, scores on the General Self-Efficacy Scale (GSES) increased, while Music Performance Anxiety Index (MPAI) scores decreased. This pattern is consistent with the notion of “Human-Centred AI that Empowers, Not Replaces” (Purwanto, 2025). For postgraduate students, confidence at this stage is often unstable. On the one hand, they are expected to form independent artistic perspectives; on the other, limited experience means that they frequently rely on external authority, such as supervisors or canonical interpretations, and may experience persistent self-doubt (Kenny et al., 2004). In traditional teaching settings, where the supervisor provides the primary or sole point of reference, students may receive clear direction, but this can also reduce opportunities for independent exploration and risk-taking (Shepherd et al., 2021). Introducing an AI-based second opinion alters this situation. Rather than functioning as an additional authority, the system operates more like an external reference that offers alternative possibilities. When students find that their initial musical decisions, for example a harmonic treatment in a Schubert song, correspond to patterns identified by the AI, this can reinforce their sense that their choices are grounded and justifiable. When differences appear, the AI output does not simply negate the student’s approach. Instead, it introduces another perspective that can be examined. This often leads students to reflect more explicitly on their own reasoning, asking why a particular decision was made and how it relates to stylistic or expressive considerations.

Such reflection appears to be central to the observed reduction in anxiety. The focus shifts away from identifying a single correct answer and towards understanding the basis of different choices. As Yan (2025) suggests, once AI moves beyond the role of a passive tool and begins to function as a socio-cognitive participant, its value lies in prompting reflection and supporting agency. In the context of vocal accompaniment, where uncertainty is inherent, this form of feedback appears to make it easier for students to articulate their views and engage more confidently in collaborative decision-making. This process aligns with Bandura’s (1997) conceptualisation of enactive mastery experience and verbal persuasion as sources of self-efficacy: even within a single session, exposure to structured external reference information may activate metacognitive processes that temporarily heighten perceived competence. We note, however, that the durability of these confidence gains beyond the experimental session remains to be established in future longitudinal research.

Several alternative explanations for the observed confidence gains should be considered. First, the increase in self-reported self-efficacy and reduction in performance anxiety may partly reflect a novelty effect: participants exposed to a new technological tool may have experienced heightened motivation or engagement irrespective of the AI’s cognitive content. Second, the absence of any external reference material in the control group means that the between-group difference may reflect the effect of receiving additional information input in general, rather than the specific epistemic function of a second opinion. Third, demand characteristics and social desirability cannot be ruled out entirely in self-report data. Future studies incorporating active control conditions (e.g., providing control participants with non-AI reference materials or peer feedback of equivalent volume) would help isolate the unique contribution of AI second opinions.

### Stability of decision-making styles and the role of AI

Although the intervention influenced confidence-related measures, no significant effect was found for decision-making style (DSI). This result is consistent with Allinson and Hayes’ (1996) dual-process framework, which treats intuitive and analytical tendencies as relatively stable characteristics shaped by long-term experience and individual differences. A single session of approximately 90 min is unlikely to produce measurable change in such traits. However, it is important to note that the null DSI result should be interpreted cautiously. As the post-hoc power analysis indicated (power = 0.51, BF₁₀ = 0.31), the study may have been insufficiently powered to detect a small but genuine effect. The finding should therefore be understood as “no strong evidence of change” rather than “clear evidence of no change.” Additionally, the DSI’s sensitivity to detecting short-term fluctuations in decision-making style in response to a single AI interaction has not been established, and this represents a methodological limitation.

Rather than indicating a limitation of the intervention, this finding helps clarify the scope of AI’s influence. The results suggest that AI does not replace or fundamentally alter how individuals approach decisions. Instead, it interacts with existing preferences. Students who tend towards intuition may use AI output as a way of confirming or articulating their impressions, while those who favour analysis may treat it as additional material for evaluation. In both cases, the underlying style remains intact. This has implications for the design of AI-supported learning environments. If decision-making styles are relatively stable, then a uniform interaction model may not be appropriate. Greater attention may need to be given to how AI systems can respond to different user tendencies, rather than assuming that a single approach will be equally effective for all learners. This perspective is in line with Krakowski et al. (2026), who emphasise that successful AI integration depends on recognising variation in human cognition.

### Theoretical contribution: constructing a new paradigm of human-AI collaborative augmentation in art education

The findings support a view of human-AI interaction that emphasises complementarity. The concept of ‘Human-AI Collaborative Augmentation’ provides a useful way of framing this relationship. It moves beyond a simple distinction between assistance and substitution, and instead highlights how different forms of capability can be brought together (Duan et al., 2019). In vocal coaching, many essential skills involve interpretation, communication, and sensitivity to context. These are not easily formalised. By contrast, AI systems are effective at processing large datasets, identifying patterns, and generating alternative possibilities (Liu and Zhao, 2024; Le et al., 2025). The interaction between these two domains appears to be where the main contribution lies. The present study provides preliminary empirical support for this model in the context of higher music education. AI second opinions do not displace students as decision-makers. Instead, they appear to be associated with conditions under which decisions are made, making it easier for students to engage with their own ideas. This observation resonates with Szostak (2025), who describes artistic innovation as emerging through ongoing interaction between human and machine perspectives. In addition, extending the concept of a “second opinion” from domains such as medicine and finance to the arts highlights its relevance in contexts characterised by uncertainty and interpretation (Abdulnour et al., 2025; Topol, 2019).

### Practical implications and future prospects

Several practical implications follow from these findings. First, the role assigned to AI in educational settings is critical. If AI is framed as a source of correct answers, it may reinforce dependency. If it is presented as a reference point, it can support independent thinking. Second, the role of the teacher may need to be reconsidered. Rather than acting solely as a source of authoritative judgement, the teacher can guide students in interpreting and questioning both their own work and AI-generated suggestions. This involves encouraging students to move beyond procedural questions and to reflect on the reasoning behind their choices. Finally, curriculum design may benefit from including explicit training in how to evaluate AI outputs. Without such guidance, there is a risk that students either accept suggestions uncritically or dismiss them without reflection. Developing a balanced form of trust, in which AI is neither over-relied upon nor ignored, may become an important aspect of professional training.

## Research limitations

Several limitations should be acknowledged. The sample was restricted to postgraduate students from three Chinese universities, which may limit the generalisability of the findings. The intervention was also short-term, and its longer-term effects, particularly in relation to performance outcomes, were not examined. The absence of an active control condition (e.g., peer feedback or non-AI reference materials) means that it is not possible to definitively attribute the observed effects to the AI second opinion specifically, rather than to the receipt of additional external input more broadly. Furthermore, the post-hoc power analysis indicated that the study was underpowered to detect small-to-moderate effects on decision-making style (power = 0.51), and the self-report nature of all outcome measures introduces the risk of common method variance. Future studies could adopt longitudinal designs and incorporate qualitative approaches to better understand how students engage with AI over time. In addition, the development of adaptive systems that respond to different decision-making styles represents a promising direction for further research.

## Conclusion

This study used a controlled experimental design to examine the impact of ‘AI second opinions’ on the collaborative confidence and decision-making strategies of postgraduate students in vocal accompaniment. The findings indicate that AI suggestions based on the “NetEase Tianyin” platform were associated with higher levels of self-efficacy and lower levels of performance anxiety, suggesting that the presence of a non-evaluative reference point can support students’ sense of agency in collaborative contexts. At the same time, no significant change was observed in decision-making style; however, given the limited statistical power for this outcome, this null result should be interpreted as preliminary rather than definitive.

Taken together, the results are consistent with the view that AI functions most effectively as a form of augmentation rather than substitution. Its role lies in expanding the range of perspectives available during the decision-making process, while leaving interpretative responsibility with the human participant. In educational settings, this suggests the importance of integrating AI in ways that encourage reflection, dialogue, and independent judgement. Over time, such an approach may contribute to the development of musicians who are able to engage with technological tools while maintaining a distinct artistic voice.

## Data Availability

The raw data supporting the conclusions of this article will be made available by the authors, without undue reservation.
